# A health impact assessment of gender inequities associated with psychological distress during COVID19 in Australia’s most locked down state—Victoria

**DOI:** 10.1186/s12889-022-14356-6

**Published:** 2023-02-03

**Authors:** Belinda M. Brucki, Tanmay Bagade, Tazeen Majeed

**Affiliations:** 1 School of Medicine & Public Health, College of Health Medicine & Wellbeing, University of Newcastle, Callaghan, NSW Australia; 2 Public Health Research Program, Hunter Medical Research Institute, New Lambton Heights, NSW Australia

**Keywords:** COVID19, Lockdown, Policy, Australia, HIA, EFHIA, Inequity, Mental health, Women

## Abstract

**Background:**

Since March 2020, when the COVID19 pandemic hit Australia, Victoria has been in lockdown six times for 264 days, making it the world’s longest cumulative locked-down city. This Health Impact Assessment evaluated gender disparities, especially women’s mental health, represented by increased levels of psychological distress during the lockdowns.

**Methods:**

A desk-based, retrospective Health Impact Assessment was undertaken to explore the health impacts of the lockdown public health directive with an equity focus, on the Victorian population, through reviewing available qualitative and quantitative published studies and grey literature.

**Results:**

Findings from the assessment suggest the lockdown policies generated and perpetuated avoidable inequities harming mental health demonstrated through increased psychological distress, particularly for women, through psychosocial determinants.

**Conclusion:**

Ongoing research is needed to elucidate these inequities further. Governments implementing policies to suppress and mitigate COVID19 need to consider how to reduce harmful consequences of these strategies to avoid further generating inequities towards vulnerable groups within the population and increasing inequalities in the broader society.

**Supplementary Information:**

The online version contains supplementary material available at 10.1186/s12889-022-14356-6.

## Background

Since March 2020, COVID19 involving a novel coronavirus (SARS-CoV-2) with rapid transmission and widespread infection brought the world to a standstill [1, 2]. COVID19 directly impacts physical health, with indirect impacts on social, psychological and economic dimensions. Consequently, numerous non-pharmacological public health interventions have been employed globally to contain and reduce disease transmission and associated deaths from SARS-CoV-2 [2–7].

**Fig. 1:**
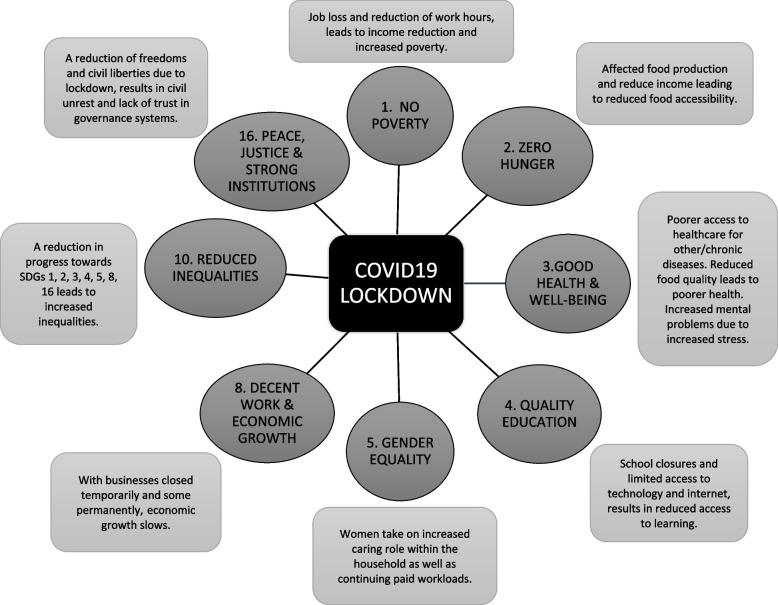
Potential impact of lockdowns on progress towards the SDGs adapted from Filho et al. [15]

Lockdown (stay-at-home, shelter-in-place) policies represent one of the non-pharmacological interventions (NPIs) enacted by governments to slow transmission through large-scale physical distancing limiting contact between people [8]. They involve differing degrees of stringency (from soft recommendations to remain at home, to more challenging orders not to leave home except with clear, limited exceptions), extend for varying amounts of time, and may be initiated at different times of the local epidemic [9]. Based on simulation studies, a rapid review found that when combined with other measures such as school closures, travel restrictions and social distancing, COVID-19 infections and deaths might reduce [5]. From March 2020, Australia’s public health response centred on the use of lockdowns, enforcing government restrictions on the movement of citizens and operation of business on a large-scale, a foreign concept to most citizens prior to then. These kinds of movement restrictions should observe public health ethics to reduce the harms resulting from them—ethics are fundamental to good public health policy. Ethical policy would maximise advancement towards the public health goal and minimise individual restriction of liberties through proportionality while reducing social injustice [10, 11]. Although numerous studies have reported on the success of lockdowns in mitigating viral transmission and flattening the curve [12–14], studies reporting on the indirect harms of lockdown are rare, as well as their contribution to non-COVID19 morbidity and mortality [14]. Previously, with other pandemics, the World Health Organisation (WHO) guidelines recommended that lockdowns be used as short-term measures for rearranging resources and protecting the health workforce [8]; however, there is no decisive and current evidence as to the best balance of measures and ethics needed to suppress a local COVID19 outbreak and reduce indirect harms. Unfair policy widens existing inequities causing further imbalance to equality, leading to downstream societal consequences such as increased poverty and hunger; education inequality; gender inequality; economic instability/recession; decreased community sustainability; health and well-being inequalities; increases in community conflict; and in the longer-term moving away from the Sustainable Development Goals (SDGs) [15]. Figure 1 shows the potential impact lockdowns can have on progress towards the SDGs, adapted from Filho et al. [15].

Physical and mental health impacts from lockdowns vary and differentially influence health directly and indirectly among different individuals and populations through all settings, widening inequities and inequalities, and causing harm at both individual and societal levels through social injustice [16, 17]. Health inequities result from systematic differences in the health outcomes of different population groups due to differences in an individual’s health position and resources arising from differing socio-economic environments [17]. Health inequities can also arise from unfair policies/interventions [17]. Table 1 shows potential health impacts (determinants) which may result from lockdowns.

**Table 1:** Potential health impacts of lockdown policies

Determinants	
**POSITIVE**	**NEGATIVE**
Reduced transmission and deaths from SARS-CoV-2 [18–20]. **(ST; LT; D)**	Economic [21–24]: Loss of job, income, business [25, 26]; occupation [27]; increase in poverty [15, 28]. **(ST; LT; D)**
Reduced infections with infectious diseases in high-risk groups such as the elderly; those with premorbid conditions/immunocompromised; healthcare workers [18–20]. **(ST; LT; D)**	Mental health: increase in affective disorders (all age groups) [21, 22, 29–36]; eating disorders (children/adolescents) [34, 37, 38]; increase in behavioural disorders (children) [39–41]; cognitive decline (elderly) [42]. **(ST; LT; I)**
Reduced incidence and deaths from seasonal influenza [43–45]; reduced incidence of other infectious diseases [46]. **(ST; I)**	Psychological wellbeing: increased stress [21, 24, 47–49]; identity loss (income/job loss) [50]; loneliness [29]; social isolation [1, 23, 24]; reduced civil liberties [51]; increase in lifestyle changes [52], loss of routine; increase in overweight/obesity [47, 53, 54]; increase in alcohol [55–57] and tobacco consumption [58]
Injury: reduced fractures (not elderly) [59, 60]; reduced emergency department attendance due to injuries [60, 61]; reduced severity of fractures due to less sport, motor vehicle accidents [60–63]. **(ST; I)**	Relationship stress: reduced social interaction [32]; social bonding [32, 64]; intimacy and sexual intimacy [64–66]; increased domestic violence [22, 67–73]; divorce [65, 66, 74]; increase in gender inequality in family and work [75–77]; family life disruptions [24, 78, 79].**(ST; LT; D; I)**
Health system: reduced health system burden due to outbreak [80–83]. **(ST; I)**	Reduced access to healthcare [84–86]: preventative screening resulting in short and long-term increases in non-communicable diseases; ceased elective surgery; ceased dental care/increased dental caries **(ST; D; I)**
Reduced lower back pain/joint pain [61] **(ST; I)**	Physical: increase in fractures in the elderly [61]; increased sleep disturbances [87]; increased screen time (children) [88]; increase in cardiovascular and metabolic disease [89–91]; increase in overweight/obesity [47, 53, 54]; increased food insecurity [22]; increase in alcohol [55–57] and tobacco consumption [58] **(ST; LT; D; I)**
Reduced premature births [92, 93] **(ST; LT; I)**	Reproductive/sexual health: reduction in HIV postexposure prophylaxis treatment [94]; increased risk of sexually transmitted infections (STI) [95]; increase in maternal and child deaths (disrupted health systems and access to food) [96] **(ST; LT; I)**
Increased physical activity [21] **(ST; I)**	Decreased physical activity [97] **(ST; LT; D; I)**
Reduced air pollution/greenhouse gas emissions due to less travel (Motor vehicles, boats, planes) [98–100]. **(ST; I)**	Increased pollution from increase in single-use items eg. takeaway food and drink packaging, increased PPE [101] **(ST:I)**

Health impacts may be direct (D) or indirect (I), short-term (ST) or long-term (LT)

Lockdowns, especially those that are less flexible, result in a significant disruption to everyday life, and consequently, many researchers have cautioned of the unintended mental health harms that may arise [14, 21, 22, 24, 29–34, 37–42, 47–51, 53–58, 64–71, 74–78, 102–109]. Psychological distress, a determinant of lockdowns and precursor to mental illness [110, 111], results from increasing uncontrollable stressors and demands, causing difficulty coping with daily life; and often triggering feelings of depression and anxiety [112, 113]. It ranges in severity, but when severe, prolonged and untreated, it contributes to the development of mental and physical illnesses such as affective and anxiety disorders, suicidality, high blood pressure and cardiovascular disease [110, 111, 114–116]. Psychological distress presents differently among men and women [117]. Poor mental health and well-being pose a greater risk for specific groups of the population [118], with strong links showing women to be more at risk when compared to men [119–122].

Research on gender disparities in mental health has shown significant correlations with gender inequalities [123]. Gender inequality refers to circumstances where individuals are consistently given different opportunities as a consequence of inequitable (avoidable and unfair) attitudes, perceptions, and social or cultural norms about gender [124–126]. It can be present in terms of health, employment, wealth, status and power [124–126]. Examples of gender inequality include lower income for similar work [126–129]; higher levels of unpaid/carer work [128]; lower rates of schooling and secure employment [127, 129–131]; increased stress [132]; less opportunity for representation in high-level jobs [126–129]; and increased risk and exposure to sexual assault, intimate partner abuse, and gender-based violence [133, 134]. Gender inequities and resulting inequalities primarily impact women and girls [126] and are linked with altered health-related beliefs and behaviours [135].

Strong support exists for assessing the health impacts of significant policies, plans, programs and projects to address inequalities [136]. An Equity-Focused Health Impact Assessment (EFHIA) is a category of Health Impact Assessment (HIA) and an essential technique to identify and evaluate inequities arising from the introduction of a policy/intervention within populations through a systematic framework incorporating health impact assessment methodology [137, 138]. The distribution of health impacts is often evaluated using existing data, information and evidence to assess the degree to which the distribution occurs due to avoidable and unfair factors to minimise these inequities and social injustice [137, 138]. Policy analysis and the identification of inequities are critical components of policy implementation. Although policies/interventions are intended to protect people from health-related harm, they inadvertently risk generating harm, worsening inequities and widening inequalities within societies [3, 139, 140]. Increased awareness of these inequities will allow policymakers to make nuanced accommodations for different populations and help to inform policy evaluation to produce a more equitable approach at state and national levels for future pandemic preparedness.

Within Victoria, Australia, in early July 2020, there was an upsurge of community outbreaks of SARS-CoV-2, and in response, on July 8, areas of Melbourne were placed into lockdown with activity restrictions increased for the remaining areas in Victoria. However, a significant decrease in viral transmission did not occur. Consequently, on August 2 2020, Victoria entered a State of Disaster and State of Emergency to enact a stringent state-wide lockdown by the Public Health Commander in conjunction with the Chief Health Officer and Premier [141]. Stay-at-home direction (No 7) was enacted [141] to restrict the movement of all Victorians, with further policy directions implemented for a proposed period of 6 weeks [141–153]. The purpose of the lockdown was to address the public health risk posed by increasing clusters of COVID19 infections through the limitation of public movement and interaction, thereby suppressing the transmission of SARS-CoV-2 to reduce infections, deaths and health-system overburden [141–150]. Stringent restrictions consistent with a stage 4 (metro)/stage 3 (regional) lockdown were imposed throughout the state, including night-time curfews and restrictions on day-time movement for activity, time, number of people and distance, both in Greater Melbourne and to a lesser degree, regional Victoria [141, 143–153]. Mask wearing was mandatory [141, 143–153]. Non-essential businesses were closed, and visitors were not permitted at private residences or aged-care facilities [141, 143–153]. The failure to observe the public health directions was punishable with penalties. The target population for the directions [141, 143–153] was all Victorians. Since the August 2020 lockdown, Victoria has endured four other lockdowns of varying durations (totalling six lockdowns since March 2020 or 264 days of lockdown), and their State of Emergency has been renewed 20 times [141, 144–171].

The gender inequities associated with increased psychological distress resulting from the Stay-at-home directions [151–171] used for COVID19 suppression and mitigation in Victoria have not yet been addressed in the literature. This study aimed to evaluate the gender inequities associated with increased psychological distress in Victorian women aged 18 and over living independently through the use of the EFHIA framework [137] during the Stay-at-home directions [151–171] for COVID19. It is hypothesised that Victorian women will experience increased psychological distress due to the gender inequities within the Stay-at-home directions [151–171], as represented by existing data and literature.

## Methods

An EFHIA was chosen due to the uncertainty about the potential, differential and significant impacts of the stay-at-home direction. This project followed a combination of the Australian Collaboration for Health Equity Impact Assessment Equity-focused Health Impact Assessment Framework [137] and the University of New South Wales Health Impact Assessment: A Practical Guide [172] and followed the standard five-step evidence-based process: screening; scoping; impact identification; assessment of impacts; recommendations [137]. Ethics approval was not needed as this study retrieved, analysed and synthesised existing published data and literature.

### Screening

The screening stage evaluated whether the EFHIA was a suitable strategy to identify the equity gaps of Victoria’s Stay-at-home Directions for 2020–2021 [141, 144–171]. Screening helped to identify the associations between policy and health, equity and inequalities in health [137] through a series of questions querying the policy’s contribution to health impacts and inequities. Supplementary Table 1 of the Supplementary Information shows an adaptation of the screening tool completed at the start of the study.

### Scoping

The scoping step established boundaries of time and scope for the assessment, determining which impacts would be considered [137]. Supplementary Table 2 of the Supplementary Information shows a checklist used to assist with the decision-making regarding the level of EFHIA to be performed. A desk-based/mini EFHIA was chosen as the timeframe for this EFHIA was particularly narrow, and there were limitations regarding capacity and resources. A steering committee was not employed as the project used a desk-based EFHIA. Supplementary Table 3 of the Supplementary Information contains a list of core values and guiding principles established and used for the EFHIA.

### Impact identification and assessment

The researchers searched evidence-based literature (BB, TM) to identify this policy's likely and possible health impacts and their effect across different population groups. The health impact and target population were determined from the evidence-based literature search. The target population for the direction [159] was all Victorians. The target population for this EFHIA was determined to be women aged 18 and over living independently, and the health impact was mental health impacts represented by increased psychological distress. A health and sociodemographic profile for Victorians was constructed using the Australian Bureau of Statistics 2016 [173] census data and the Department of Health and Human Services Victorian Population Health Survey 2016 [174]. Thematic mapping was performed to help establish the determinants and their causal pathways to psychological distress and mental illness.

An extensive literature review involving a review of quantitative and qualitative published studies and grey literature was undertaken to find evidence of the relationship between gender and psychological distress and the psychosocial determinants identified in the study during lockdown; using the search terms and combinations provided in Supplementary Table 4 of the Supplementary Information. Sources of information and methods used to obtain the information are given in Supplementary Table 5 of the Supplementary Information. The project included studies published until December 2021. All studies included for analysis were published in English and the first six pages of each search result were reviewed for analysis. The Impact Assessment Matrix [172] provided the framework to analyse and synthesise the evidence. Supplementary Table 6 of the Supplementary Information shows the completed Impact Assessment Matrix. Published peer-reviewed academic publications and local-government health data were weighted with greater significance than grey data by the researchers (BB, TM). Data were analysed, and the impacts were synthesised. Impacts were classified as moderate or limited, positive or negative, highly probable or probable, and long or short term.

## Results

Results from the demographic profiling on the 2016 census [173, 175], show Victoria recorded 5,926,624 people and of these, 50.9% (*n* = 3,018,549) were female and 49.1% (*n* = 2,908,077) were male [173, 175]. The total population over the age of 19 is 4,489,371 persons. The median age of Victorians is 37 years, and with over 124 ethnicities, Victoria is considered a highly multicultural state [173]. 13% of Victorian households do not have internet access [173]. Throughout the remainder of this paper, the authors have tried to maintain consistency in language regarding sex and gender but original data sources are inconsistent, and so to stay inline with original sources, we refer to either male/female or men/women interchangeably. We realise, however, that they are different constructs.

Table 2 compares Victorian males and females socio-demographically [175]. It shows a higher proportion of females (55.7%) out of the workforce and unemployed (6.7%) compared with males (28.1%, 6.6%), and a more significant proportion with part-time jobs (48.3% vs 22.2%) [175]. The table shows that females are more likely to spend time in caring roles and unpaid work (domestic; care of children, disabled, sick or elderly) than males [175]. The top employment industries for females are healthcare, education and retail [175]. The top employment industries for males are construction, manufacturing and retail [175].

**Table 2:** Victorian (2016) indicators for employment, education and unpaid work by sex (**Source**: What we do? Employment, Unpaid work: Informed Decisions Community Demographic, Available from https://profile.id.com.au/australia/employment-status?WebID=110) [175]

	**Victoria 2016** **Aged 15 and over**			
	**Males**		**Females**	
Employed Full-time	**69.3%** *n* = 1,067,764 *N* = 1,541,195		**43.4%** *n* = 602,791 *N* = 1,388,404	
Employed Part-time	**22.2%** *n* = 1,440,119 *N* = 1,541,195		**48.3%** *n* = 670,577 *N* = 1,388,404	
Unemployment rate	**6.6%** *n* = 101,076 *N* = 1,541,195		**6.7%** *n* = 92,393 *N* = 1,388,404	
Total Labour Force participation	**65.5%** *n* = 1,541,195 *N* = 2,353,502		**55.7%** *n* = 1,388,404 *N* = 2,492,205	
Persons not in Labour Force	**28.1%** *n* = 662,274 *N* = 2,353,502		**38.0%** *n* = 947,862 *N* = 2,492,205	
Unpaid Domestic work	**63.8%** *n* = 1,502,166 *N* = 2,353,506		**72.8%** *n* = 476,051 *N* = 2,492,194	
Unpaid care disability, elderly, long-term illness	**9.4%** *n* = 220,774 *N* = 2,353,503		**13.6%** *n* = 340,093 *N* = 2,492,206	
Unpaid care children	**23.7%** *n* = 558,178 *N* = 2,353,497		**30.9%** *n* = 770,988 *N* = 2,492,209	
Top 3 Employment Industries	**13.9%** *n* = 200,807 *N* = 1,440,104	Construction	**20.9%** *n* = 270,795 *N* = 1,296,021	Healthcare & Social assistance
	**10.5%** *n* = 151,724 *N* = 1,440,104	Manufacturing	**12.8%** *n* = 165,409 *N* = 1,296,021	Education & Training
	**8.5%** *n* = 122,391 *N* = 1,440,104	Retail Trade	**12.1%** *n* = 157,246 *N* = 1,296,021	Retail Trade

Results from the health data profiling show that for females within Victoria, a mental health condition was the most common long-term health problem, while for males, it was asthma [175]. Table 3 shows that in 2016, females were experiencing higher levels of high to very high psychological distress than males [174, 175]. It also shows females to be more likely (28.7%) to experience anxiety or depression than males (20.0%) [174, 175]. In 2020 pre-pandemic, 57% (*n* = 1,140; *N* = 2,000) of Victorians felt socially disconnected [176]; while in 2018, 25% (*n* = 419; *N* = 1,678) of Victorians felt lonely [177].

**Table 3:** Proportions of psychological distress, anxiety and depression experienced by males and females in Victoria in 2016 (**Source:** Department of Health and Human Services. Victorian Population Health Survey 2016 [174])

	**Victoria 2016**	
	**Males**	**Females**
Psychological Distress (High/Very high) *18 yrs & over* [174]	**13.2%** *n* = 383,866 *N* = 2,908,077	**16.5%** *n* = 498,060 *N* = 3,018,549
Anxiety/Depression *18—84 yrs* [174]	**20.0%** *n* = 581,615 *N* = 2,908,077	**28.7%** *n* = 866,234 *N* = 3,018,549

**Fig. 2:**
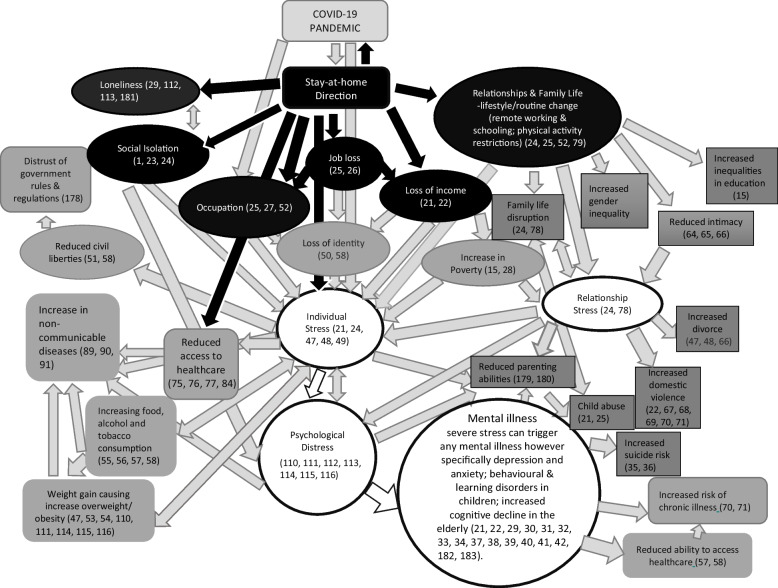
Thematic mapping demonstrating hypothetical causal pathways of psychosocial determinants contributing to psychological distress during lockdown

The screening step enabled the identification of the health impact and psychosocial determinants. The health impact identified as a highly likely impact of lockdown was psychological distress. From screening, psychosocial determinants directly impacting lockdown were loneliness, social isolation, occupation, income, and relationships/family life. Figure 2 shows the thematic mapping resulting from the scoping step of the EFHIA framework [137]. Thematic mapping assisted in the identification of causal links from the psychosocial determinants to the mental health impact of psychological distress.

Within the diagram, individual stressors and relationship stressors directly impact on psychological distress, which directly impacts on mental illness (white circles and arrows). Individual stress can also impact families and relationships, resulting in relationship stress and contributing to psychological distress [24, 78]. All psychosocial determinants within this analysis that are a direct result of the lockdown, are in black circles and are designated by a black arrow approaching them. Causal pathways from the psychosocial determinants of lockdown, were drawn to show the impact of individual stress and psychological distress from ongoing individual stress [110, 111]. Psychological distress can be experienced directly and indirectly from the psychosocial determinants [114, 115].

### Loneliness

Loneliness, a subjective feeling of disconnectedness, has been associated with increased mental health problems such as stress, psychological distress, depression, suicidal ideation and cognitive decline, as well as physical health problems such as cardiovascular disease and premature mortality; through increased involvement with health-risk behaviours [104, 169, 178–186]. Within the context of COVID19 lockdowns, loneliness has been highlighted as one of the significant determinants of depression, anxiety and psychological distress [187, 188].

### Social isolation

Social isolation or disconnectedness refers to an impartial physical separation from social connections [189]. Brief encounters with social disconnection can trigger negative emotions, while prolonged disconnection is linked to the development of internalising disorders such as depression and suicidality [189]. Lockdowns directly result in a restriction of mobility affecting the social connection of people [176, 190].

### Occupation

Research has recognised certain occupations to be associated with a greater risk of psychological distress [191, 192]. Occupations considered frontline or essential in Australia for COVID19 have included those in security, hygiene, healthcare, essential retail, transport and delivery, childcare and education, aged care, disability and law enforcement [173, 193]. Workers within these occupations must contact the public directly, putting theirs and their family’s health and safety at risk when returning home [193]. Healthcare was used in this assessment.

### Income

A strong association has been observed between an individual’s income and mental health [194]; low income, job loss, unemployment and poverty resulting in financial strain, psychological distress, and mental illness [116, 195–199]. During COVID19, there has been a high prevalence of psychological distress in people who have lost their jobs or casual workers who have no income during lockdown [196]. Numerous studies have highlighted the impact of socioeconomic stress (including job or income loss) from lockdown on individuals and its contribution to psychological distress [21–24, 200].

### Relationships and family life

Relationship dissatisfaction is strongly associated with psychological distress for both men and women [201]. Individuals with good relationship quality showed better mental health and performed significantly better on mental health scales than individuals with poor or no relationship quality [32]. Poor mental health affects individuals and the network of people with close involvement, such as relationships with partners and children [201–204]. Parent mental health directly affects parenting ability, with continual negative emotions triggering children’s emotional, behavioural and learning problems [205–208].

### Impact assessment

Impacts were evaluated using locally available data for Victoria and Australian data to assess whether a gender disparity exists for women regarding psychological distress during the lockdowns using the psychosocial determinants identified during screening. Table 4 briefly describes the key local data sources used in this assessment.

**Table 4:** Main local data sources used to inform this assessment with brief descriptions

Data Source	Description
**VicHealth Coronavirus**[176] VicHealth Coronavirus. Victorian Well-being Impact Study	• Impact on health and well-being of COVID19 • *N* = 2000 Victorian adults completed online population-based survey during the first lockdown in March • Study characteristics—self-reported, population-based, online, cross-sectional, volunteers
**The COLLATE project**[200, 209] COvid19 and you: mentaL heaLth in AusTralia now survEy	• A series of monthly online population-based surveys (*N* = 5158) tracking the impact of COVID19 and government restrictions on the mental health and well-being of Australians conducted by Swinburne Centre for Mental Health (data collected from the first wave of COVID19, not always lockdown specific depending on the month) *n* = 1292 of the sample surveyed with self-reported mood disorders • Study characteristics—self-reported, population-based, online, cross-sectional, volunteers
**ANU survey**[210] Tracking outcomes during the COVID19 pandemic	• Monitoring the impacts of COVID19 • population-based surveys (April 14/4–27/4 N = 3155), May (12/5–24/5 *N* = 3249), on Australian population and August (10/8–24/8 *N* = 3061) for Australian & Victorian data • Aims to update national-level trends in well-being data • Study characteristics—survey used is Life in Australia_TM_—longitudinal survey of a large, representative sample of Australians
**ABS surveys **[211–220] Household Impacts of COVID19 surveys	• Monthly household impacts of COVID19 online surveys on the broader Australian public (not always lockdown specific depending on the month), *N* = 1,000 per month • Study characteristics—self-reported, population-based, online, cross-sectional, volunteers

The VicHealth study [176] showed that 16% of the population reported an increase in psychological distress to high levels during Victoria’s second lockdown in 2020. Psychological distress was more evident in 18–24 year-olds; respondents in inner metro areas; respondents who speak another language at home; people with disability; unemployed respondents; and those living in bushfire areas [176]. Gender differences were not observed in this study.

ABS surveys [211–220] examined mental well-being in the Australian population during the first lockdown in 2020 and reported poorer results than before the lockdown. Table 5 shows higher levels of anxiety and depression for females when compared with males. Data further suggested that from May to August 2020, 19% of females compared to 9% of males felt so depressed that nothing could cheer them up [219].

**Table 5:** Anxiety and depressive symptoms recorded (**Source:** ABS Household Impacts of COVID19 survey August, *N* = 1,000 [219])

Anxiety-based symptoms	Females (%)	Males (%)
Restless or fidgety	43.5	38.4
Nervous	50.0	41.0
Everything an effort	44.7	36.2
Symptom relating to depression—Loneliness	28.0	16.0

Biddle et al. [210] found psychological distress to increase during the first lockdown, with 47% of the survey sample indicating they were more stressed even when infection numbers decreased. Other elevations in psychological distress that occurred between May and August, saw a considerable deterioration of mental health for females in Victoria during the second lockdown [210]. A strong association was evident between a symptom of depression (loneliness) and social connectedness with increased stress due to socioeconomic factors, such as income, housing and work hours [210]. Similarly, data from the COLLATE study [200] showed that during the first lockdown, negative emotions such as anxiety, depression and stress were more elevated for women.

Data from newspaper reports showed Lifeline in Victoria recorded a 30% increase in telephone counselling from the start of lockdown 2 Stage 4 restrictions due to increased stress and anxiety arising from social distancing, quarantining, isolation and disconnection from family and friends [221]. Headspace saw a 50% increase in young people with an increased risk of self-harm and suicide who had been admitted to the emergency department with a mental health crisis. Referrals for young people to the emergency department for self-harm increased 33% compared with August 2019 [221]. There was a significant increase in the need for mental health services seen among women presenting with anxiety, depression and obsessive–compulsive disorder at The Alfred hospital [222], with new referrals for women increasing from 5 per week in 2019 to 110 within one week in late July 2020.

**Fig. 3:**
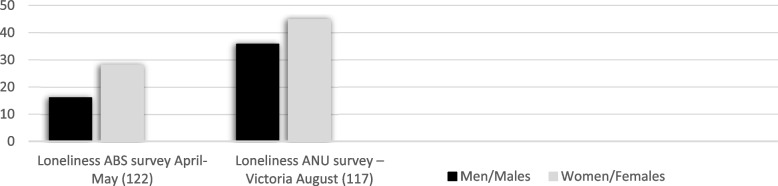
Loneliness ratings for Men and Women in Australia, April/May and Victoria, August 2020 (**Source:** ABS Household Impacts of COVID19 survey 29/4/2020 -4/5/2020 N = 1,000 [215] & Tracking outcomes during the COVID19 pandemic 2020, *N* = 3,061 [210])

The psychosocial determinant, loneliness, was assessed using ABS survey data [215] and ANU survey data [210]. In Fig. 3, both surveys showed an increase in loneliness for both men and women over the months; however, even more elevation for Victorian women in August 2020. A strong association was evident for women for psychological distress with a symptom of depression (loneliness), with increased stress due to socioeconomic factors, such as income, housing and work hours [210]. Lifeline and Beyond Blue data for telephone counselling show increased loneliness (Lifeline, Beyond Blue) from April–May 2020 with no note of gender differences [223, 224].

Data from the VicHealth study [176] for the psychosocial determinant, social isolation, showed that 30% of respondents found it harder to stay connected to others, with a 37% decrease in feeling connected with others and a 23% increase in social isolation. Respondents with a disability, living in regional areas, unemployed, low income, living alone or in a share house, reported feeling even less socially connected; however, no gender difference was observed [176]. A strong association was evident for women for psychological distress with social connectedness, with increased stress due to socioeconomic factors, such as income, housing and work hours [210].

Healthcare worker data was used for the determinant, occupation. Infection analyses were conducted on the Healthcare Worker Dashboard [225] for September and October 2020 and showed that for healthcare workers, infection was significantly higher than non-health-care workers, with odds ratios of 5.02 compared with 1. The odds ratio was highest for aged care workers at 11.81 [225]. Data from the Department of Health and Human Services (2021) showed that within the healthcare industry, the second lockdown in Victoria saw higher numbers of infections among healthcare workers [226], as shown in Table 6. Between July 1 to August 25, 2020, 69 -90% of healthcare worker infections were acquired at work [227].

**Table 6:** COVID19 infections in a healthcare setting (**Source**: Healthcare Worker Infections Dashboard 2021, Available from: https://healthcareworkersaustralia.com/analytics/) [227]

Occupation	Total number of COVID19 infections	% COVID19 infections acquired at work
Aged and disability care workers	924	90
Medical practitioner	106	77
Nurse (all setting)	922	89
Other healthcare areas	303	69

Data from the Alfred Hospital showed an increase in anxiety presentations from healthcare workers [222], while the Royal Melbourne Hospital shows that 68.3% of infected nurses work within geriatric and rehabilitation wards [228].

Income data from Equity Economics (2020) showed that between February and July 2020, women lost 61% of their jobs [229]. During the second Victorian lockdown, industries employing 243,800 women and 210,000 men closed [229]. Since March 2020 within Victoria, throughout the first and second lockdowns, the ABS recorded a steady decrease in payroll data for women in jobs of 7.1%, with July data (before the second lockdown) showing job loss for women to be five times the rate for men [230, 231]. Within Australia, ABS data from March—April 2020 showed employment fell by 5.3% for women and 3.9% for men [231]. Within Australia, from March 2020, the most burdened industries by job loss were accommodation/food services, retail and arts/recreation [230–232]. Australian data describing hours worked showed men dropped 7.5% while women dropped 11.5%, consequently burdening women more so than men [232]. The COLLATE study [200] found that financial stress and job loss were associated with increased psychological distress during lockdown, while lower levels of distress were associated with higher incomes and savings.

For the relationships/family life determinant, 20% of relationships within Victorian homes became more strained during lockdown, and this was particularly apparent for groups who were unemployed; parents with child (ren); or those in a share house [176]. Table 7 shows the burden and increased stress placed on home life with lockdown. From the table, it is evident that mothers (women) spend significantly more time helping children, looking after children, carrying out domestic work, and other caring work than fathers (men) do.

**Table 7:** Burden placed on Mothers and Fathers for homelife factors during COVID19 lockdown (**Source**: VicHealth Coronavirus. Victorian Well-being Impact Study, *N* = 2,000 [176]& ABS. Household impacts of COVID19 survey. 6—10 July 2020, *N* = 1,000 [220])

Homelife	Mothers (%)	Fathers (%)
Primary responsibility for looking after preschool children during lockdown	76.0 [176]	8.0 [176]
Most time spent helping children with remote learning	72.0 [176]	26.0 [176]
Unpaid domestic work	80.0 [220]	39.0 [220]
Unpaid caring	38.0 [220]	11.0 [220]

A study by Relationships Australia [74] showed that 42% of Australians experienced an adverse change in their relationship due to lockdown, with 55% reporting socioeconomic reasons for change. No gender difference was reported in this study. However, an Australian study [75] showed that paid work time was slightly lower and unpaid work much higher for mothers during lockdown than before it, with fathers noticing a slight increase in time spent caring for children, and most mothers noting an increase in dissatisfaction.

Supplementary Table 6 of Supplementary Information contains the Impact Assessment Matrix used in the analysis of the studies to demonstrate the level and strength of the evidence supporting the impacts and determinants of lockdown. The table shows that when this assessment was performed, limited local data were available; however, of all the evidence analysed, a moderately strong relationship was found between women’s gender inequities and the increased psychological distress resulting from lockdown policy. Similarly, the psychosocial determinants of loneliness, income, occupation and relationships/lifestyle were found to also increase psychological distress in women with moderate strength. Social isolation demonstrated limited strength. The nature of the impact is negative, and the potential size of the impact is large. This impact can have short and long-term effects.

## Discussion

This study evaluated the gender inequities associated with increased psychological distress resulting from the Stay-at-home directions [151–171] used for COVID19 in Victoria during 2020—2021 using the EFHIA framework [137]. It highlights avoidable inequities which contribute to mental illness. The evidence gathered supported the hypothesis: a gender disparity was identified for women for the mental health impact of increased psychological distress resulting from lockdown policy. The psychosocial determinants—loneliness, income, occupation and relationships/family life were found to contribute to increased psychological distress for women in ways which could have been avoided.

The results showed moderately strong support for the impact of increased psychological distress. Data for Victoria and Australia obtained from the Tracking Outcomes during the COVID19 Pandemic study [210], ABS Household Impacts of COVID19 surveys [211–220] and COLLATE project [200, 209] all show an increase in psychological distress that is greater for women when compared with men. However, data from the Victorian Well-being Impact study [176], Lifeline [221, 224], and Headspace [221] did not demonstrate a gender difference for psychological distress. These results may be due to small sample sizes or the time-point in which the sample was taken. Extensive evidence was found in the literature supporting increased psychological distress during lockdown for women, with women experiencing higher levels of distress than men [21, 22, 30, 31, 33, 55, 103, 233–253], and with some studies indicating that women were predisposed to experience higher levels [21, 31, 33, 122, 254] due to higher baseline levels in non-pandemic conditions. Table 3 is consistent with higher baseline levels of psychological distress, anxiety and depression for women compared to men. Xiong, Lipsitz [109] reviewed the association between the COVID19 pandemic and mental health for 19 cross-sectional studies and found women to be associated with higher levels of mental distress when compared with men. Psychosocial factors highlighted to be important in understanding the distress include age, gender, physical security, income, work conditions and work [21, 22, 33]. Consequently, the results suggest that pre-existing gender inequity exists for women’s mental health and lockdown policies most likely exacerbated this inequity.

Data for Victoria and Australia show income changes during lockdown disproportionately burdened women. More women became unemployed or represented among the part-time workforce [231]. Women’s paid hours of work decreased the most compared to men [232]; for some, due to the increased need to be carers during lockdown [77, 255]. This uneven job and income loss resulted in increased financial stress for women. Women are more likely to be employed in the casual or part-time workforce compared with men, causing them to have fewer leave entitlements [230]. Government policies introduced within Australia to support income loss through lockdown did not support many women in various industries [230], as work for them is often less secure and lower paid [256]. Global studies support women’s income loss to be disproportionately affected by lockdown [76–78, 257–259]; however, studies also suggest that women’s increased need to be carers at home during this time may contribute to this [77, 255]. Consequently, lockdown reinforced a reduction of paid work and increased unpaid work for women [260].

Table 2 shows the top three industries for employment for women are healthcare/social assistance, education/training, and retail, classed as essential services during the pandemic, thereby leaving women disproportionately exposed to increased stress during lockdown from high-pressure and high-risk work. Workers in these industries were at higher risk for infections and could not work from home during lockdown [175, 227, 261, 262]. Increased mental health presentations for healthcare workers in Victoria demonstrate the increased distress and anxiety experienced due to increased infections experienced by healthcare workers [222, 228]. Evidence of increased distress and anxiety is noted in the global literature [263–275]. During the SARS and MERS epidemics, increased stress, anxiety, depression, and psychological distress were seen in healthcare workers, with some studies showing persisted elevation one year post the epidemics [276–283]. Increased anxiety and stress in healthcare workers is partially due to increased infections which have resulted from inadequate personal protective equipment [284].

In Victoria, relationships/family life were shown to become more strained [176]. Pre-pandemic data (Table 2) demonstrated that women were primarily responsible for unpaid work, whether domestic duties or the care of children, elderly family, sick or disabled. With 13% of Victorian households without internet access [173], working from home and home-schooling became impossible for these families, contributing to increased stress. Similar data can be seen in Table 7 during lockdowns. Within the literature, lockdowns were consistently shown to reduce paid work and increase unpaid work for women [75, 220, 260]. Mothers were more adversely affected by home-life stress [24, 41, 76, 78, 258, 285–288] and parental stress due to the uneven division of the care burden [109, 242, 246, 260, 289–295]. Factors contributing to increased parental stress included reduced parent resilience, social connections, sole parents, having special needs children and younger children [182].

The lockdown determinant of loneliness also demonstrated increased psychological distress disproportionately for women. Data from Victoria showed increased loneliness for both men and women from the first lockdown in April–May to the second in August 2020, with women scoring higher on both occasions [210, 215]. Another study in Victoria showed a strong association for loneliness between women and psychological distress due to socioeconomic factors such as income, housing and work hours [210]. Pre-pandemic [296, 297] and post-pandemic global studies further confirm that loneliness is a higher risk factor for women than men [1, 47, 242, 244, 245, 285, 296–309].

Conversely, the direct determinant of social isolation was not found to contribute to increased psychological distress for women, even though social isolation is a direct result of the restriction of mobility and connectedness of people that occurs with lockdown [176, 190]. Pre-pandemic studies have found men to be more socially isolated than women [310–313], and the ANU study [210] found women to feel more connected than men. Global studies during the pandemic have shown mixed results regarding social isolation [189, 246, 314–320] with no clear association with women experiencing more significant amounts of social isolation during lockdown. Consequently, social isolation may be a more suitable mental health determinant for men during lockdown.

It is well known that social factors affect mental health and the risk for mental illness [321]. Gender, a social construct, is considered a structural and social determinant of mental health/illness [117, 322–325]. The results of this study demonstrate that increased gender disparities are evident in women’s mental health with the use of lockdown policies in Victoria from 2020–2021. Differential vulnerability and exposure to risks and differences that impact mental health and the outcomes, are influenced by a person’s gender [325], and in this EFHIA, women experience poorer outcomes. The lockdown determinants used in this study further suggest that gender differentially affects the control and power both men and women have over these psychosocial determinants. Unfair public health policy that is negligent of mental health not only predisposes women to longer-term stress and distress but increases the risk of mental illness and poorer physical health outcomes [111, 114–116], creating additional levels of injustice, particularly during a pandemic. It is quite probable for the Victorian lockdowns, the compounding effects of multiple lockdowns over time, would worsen the determinants contributing to psychological distress and the risk of long-term mental illness [326].

In order to address the problems of increasing mental illness during COVID19, improved awareness of the gender dimension of mental health during lockdowns is required. Although this study has addressed a gap within the literature regarding policy generating gender disparities in mental health during lockdowns; future research is critical to address others, especially with the increased risk of future pandemics arising from the ecological spillover from animals to humans and environmental damage [327]. Based on our findings, we recommend that future policy and decision-making prioritise minimising negative impacts and injustice so that they may better reflect public health ethics.

### Limitations of the assessment

Limited local data was available at the time of the assessment; therefore, studies with Victorian or Australian data were selected for local data. Most studies use population-based surveys where people volunteered to participate and self-report responses, introducing response bias and sampling errors. Sample sizes were often small; methods were not always detailed; consequently, data may not be generalisable. Samples were often cross-sectional, being, restricted to a specific time-point, which limited the evaluation of the long-term impacts on mental health. In most of these studies, sampling was conducted during the early stage of COVID19 and lockdown in April 2020; therefore, mental illness will not have become established.

Further limitations involve the framework used to assess the equity deficit. A mini EFHIA generally evaluates the existing literature and data by a single researcher. A comprehensive EFHIA, incorporating a focus group of key community stakeholders, could help reduce bias, enabling an improved selection of determinants for the equity analysis.

### Future research

Future research should endeavour to understand further the factors contributing to stress and mental illness during lockdown to mitigate the avoidable mental health inequities attributable to public health lockdown policies used during COVID19. A comprehensive EFHIA incorporating the use of a focus group of key community representatives would be a helpful next step to elucidate further the inequities associated with these Stay-at-home directions [151–171] and reformulate policy for future pandemic preparedness.

Future research should also look to characterise the women affected differentially by lockdown policy further. Evaluating the social drivers can help further understand the impact of inequities and inequalities of policy on women. Additional studies that aim to fully elucidate the complex dynamics of psychological distress and the development of mental illness are needed. By understanding these factors, we can better understand the drivers of mental health inequities and inequalities within policy. Research in this area would also help to understand how this hinders progress towards the Sustainable Development Goals.

## Recommendations

The following recommendations are suggested.

### Upstream

1. Educating and supporting families and couples upstream through evidence-based multimedia education programs aimed at changing gender-based norms perpetuating the inequities of homelife and parenting for women [222].
2. Developing national and state-level income relief policies addressing the social and economic policies that continue to drive inequalities and provide sufficient relief to allow workers to stay home without income stress [222, 230].
3. Research has shown that animal ownership can be beneficial in mitigating some of the detrimental mental health effects of lockdown [328–331]. Animal shelters could initiate a borrowing service to assist people’s well-being for those who would benefit from having an animal but may be unable to commit to a pet as a long-term endeavour.
4. Pre-pandemic data shows that being outdoors is associated with increased positive emotional well-being with the potential to mitigate feelings of loneliness [332–334]. Lockdowns restricting time spent outdoors should be discouraged as the ability to spend time outdoors becomes even more important to mental health and well-being [335]. Developing policy informed by this data may affect emotional well-being during future surges or pandemics.
5. School and childcare closures create additional burdens for parents, predominantly women. Although children were initially thought to be vectors for SARS-CoV-2, data to date lacks evidence of widespread paediatric transmission [336, 337]. When formulating policy, policymakers should consider the balance of risks to children’s health, development, well-being and learning generated by not attending school versus disease transmission [338–340]. They should also consider the effect closures will have on the family unit. Lockdown policies that limit the closure of schools and childcare are critical in reducing the burden of unpaid work, particularly for women and improving women’s mental health. These policies should also allow for nuance to include families with medical vulnerabilities [341–344].

### Downstream

1. Providing increased financial accessibility to mental healthcare through increased Medicare rebates for mental health sessions for all individuals, with additional sessions aimed at improving women’s mental health [345–347].
2. Ensuring increased capacity within healthcare with cultural and gender diversity to effectively manage increased demand for mental healthcare [118].
3. Ensuring increased accessibility to mental health care by removing gatekeeping and enabling individuals to make direct contact with their mental health provider without the referral of a primary care practitioner to reduce waiting times. Gatekeeping is traditionally associated with a need to control healthcare expenditure. Although gatekeeping has been associated with better quality of care, it is also associated with lower healthcare use and patient satisfaction [346–348].
4. Supporting families and relationships during the pandemic through family and relationship therapy with professionals trained in managing lockdown effects prevents the snowballing effect of increased stressors [286].

## Conclusion

The EFHIA framework helped to identify inequities associated with gender and a precursor of mental health problems, psychological distress, for the lockdown policies used in Victoria during 2020–2021. It provides an important perspective to the existing literature, highlighting areas where public health policy can be modified to reduce gender inequities and inequalities. Literature suggests that progress towards SDGs, including gender equality, will be obstructed by lockdown policies however further evaluations should be pursued as evidence. Public health practitioners should work closely with policymakers through the identification of key strategies to improve social justice in implemented policies. With increased risks of future pandemics due to ecosystem and climate change, understanding the impacts of lockdown policy can help prepare us to reduce inequities in future lockdown policy, consequently the importance of this work reaches beyond the scope of COVID19.

## Supplementary Information

**Additional file 1: Supplementary Table 1. **Screening questions used during the screening phase of the Equity Focused Health Impact Assessment(adapted from Appendix 1: Screening Tool for Health Impact Assessment, Health Impact Assessment: A Practical Guide (1)). **Supplementary Table 2.** Checklist for level of depth of HIA (reproduced from Appendix 2: Checklist for level of depth of HIA, Health Impact Assessment: A Practical Guide (1)). **Supplementary Table 3.** Core values and guiding principles. **Supplementary Table 4.** Search terms and combinations used to find evidence. **Supplementary Table 5.** Source of information and methods used to obtain it. **Supplementary Table 6.** Impact Assessment Matrix (reproduced from Appendix 3: Comprehensive Assessment Matrix, Health Impact Assessment: A Practical Guide (1)).

## Data Availability

The original contributions generated for this study are included in the article and supplementary material, further enquiries can be directed to the corresponding author.

## Abbreviations

EFHIA: Equity Focused Health Impact Assessment
HIA: Health Impact Assessment
NPI: Non pharmaceutical intervention
SDGs: Sustainable Development Goals
ABS: Australian Bureau of Statistics
