# Supplementary material for: A health impact assessment of gender inequities associated with psychological distress during COVID19 in Australia’s most locked down state—Victoria
Source: BMC Public Health. 2023 Feb 3;23:233. doi: 10.1186/s12889-022-14356-6 (PMC9894749; doi:10.1186/s12889-022-14356-6)
Supplement: Supplementary file 1 — Additional file 1: Supplementary Table 1. Screening questions used during the screening phase of the Equity Focused Health Impact Assessment(adapted from Appendix 1: Screening Tool for Health Impact Assessment, Health Impact Assessment: A Practical Guide (1)). Supplementary Table 2. Checklist for level of depth of HIA (reproduced from Appendix 2: Checklist for level of depth of HIA, Health Impact Assessment: A Practical Guide (1)). Supplementary Table 3. Core values and guiding principles. Supplementary Table 4. Search terms and combinations used to find evidence. Supplementary Table 5. Source of information and methods used to obtain it. Supplementary Table 6. Impact Assessment Matrix (reproduced from Appendix 3: Comprehensive Assessment Matrix, Health Impact Assessment: A Practical Guide (1)). [file 12889_2022_14356_MOESM1_ESM.pdf]

<u>Supplementary Table 1: Screening questions used during the screening phase of the Equity Focused Health Impact Assessment</u> (adapted from Appendix 1: Screening Tool for Health Impact Assessment, Health Impact Assessment: A Practical Guide (1))

Covid-19 Victorian Lockdown Public Health Order July 2020 onwards

Target Population – all residents in the state of Victoria

1. ***What is the proposal about?***
   A suppression strategy aimed at slowing the transmission of SARS-CoV-2 to prevent unnecessary deaths & hospital overflow.
2. ***What is the context outlined for the proposal? (eg. policy context, history)***
   The emergence of a novel communicable disease causing a pandemic, and increasing case numbers of community transmission triggered the deployment of suppression strategies such as lockdown or stay-at-home orders.
3. ***Does the proposal concern any of the following determinants?***
   - ✓ Lifestyle
   - ✓ Physical environment
   - ✓ Social/economic environment
   - ✓ Capacity of the health system to impact on these determinants
   - ✓ Other – individual psychological environment determinants such as loneliness & social connectedness
4. ***What are the assumptions embedded in or underpinning the proposal?***
   By locking down cities/states transmission will slow reducing health-system burden & fatalities.
5. ***Why does this proposal have potential to impact on health?***
   The nature of this proposal is directly about protecting the population's health from contracting a novel communicable disease.
   What are the:
   Potential Positive Impacts
   Decreased disease prevalence, infections, deaths & health-system burden.
   Potential Negative Impacts
   Loss of jobs/business/income; increased stress/distress, mental illness & speed of cognitive decline in the elderly with dementia in aged care.
   Intended consequences
   Decreased infections, deaths & health-system burden; flatten the curve
   Possible unintended consequences
   Increased stress & emergence of mental illness; increased noncommunicable disease (increased smoking, alcohol consumption, weight gain); increased job loss, poverty leading to economic recession; increased domestic violence & divorce; gender inequality; education inequities. Decreased risk; incidence, morbidity, mortality from seasonal flu & other communicable diseases; decreased environmental pollution.
6. ***Describe any information which identifies the nature and extent of the impacts on health for this type of proposal.***
   Previous pandemic research, COVID research; any previous HIAs; WHO guidelines; current research; census data ABS; Australian health data; Victorian health data; media releases; newspaper articles; Victorian State financial data sets.
7. ***List the groups most likely to be affected by this proposal.***
   Low SES, income; homeless; mentally ill; women; young families; individuals with disabilities (physical/cognitive); CALD; poor living &/or working conditions; Indigenous Australians; the elderly; immunocompromised; people with chronic health problems or who are pregnant.
8. ***What are some of the potential equity issues?***
   Desirable
   Reduced risk and incidence of COVID infection; seasonal influenza & other communicable diseases; reduced pollution cars, boats; planes
   Undesirable
   Access to money, work, education, adequate shelter, transport, technology, healthcare. Gender inequities; mental health inequities; age discrimination; CALD inequities; SES inequities; education inequities.
9. ***Is a HIA appropriate? Why or Why not?***
   A desk-based EFHIA (Equity-focused health impact assessment) will help to explore the inequities emerging from lockdown COVID-19 suppression strategies/policies to inform and strengthen future pandemic preparedness plans.
10. ***Checklist summary***
    The policy directly & indirectly will potentially affect health. It is unclear what potentially serious negative impacts might result, but further investigation to elucidate this is warranted. As this is a fairly novel policy, the potential health impacts are not well known, but unlikely to be minor, so it may not be straightforward to suggest effective ways to maximise beneficial and minimise harmful effects.

    The entire population of Victoria will be affected by the policy including all vulnerable, socially excluded & disadvantaged groups. There are community concerns about the impact of the policy.

    The extent of the disruption to population is likely to be major with potentially high costs through various avenues.

    Due to limited time & resources, & considering there is no potential to change the current policy, the use of a desk-based EFHIA can highlight need for a comprehensive EFHIA to advise policymakers & improve policy for future deployment.

    Recommendations/comments
    Proceed to desk-based EFHIA. No known HIA has been conducted on this proposal.

Supplementary Table 2: Checklist for level of depth of HIA (reproduced from Appendix 2: Checklist for level of depth of HIA, Health Impact Assessment: A Practical Guide (1))

ISSUE	QUESTION	RESPONSE TO QUESTION	GUIDANCE ON THE APPROPRIATE LEVEL	MORE/LESS COMPREHENSIVE	DEPTH DECIDED
Scale of the proposal (eg. type, topic, investment)	Are the size and importance of the proposal significant?		The greater the size and importance, the more comprehensive the HIA should be.		
Significance of health, based on screening	Are there significant potential health impacts of the proposal?		The greater the significance of potential health impacts, and the higher the degree of uncertainty the more comprehensive the HIA should be.		
Timing	How urgent is completion of the HIA to influence decisions?		If there is relatively high urgency then select a less comprehensive HIA.		
Timing	Is the timing critical in relation to other policies/programs/projects /issues?		If timing is critically linked to other policies/ programs/projects developments and timeframes are short, elect a less comprehensive HIA.		
External interest	What is the level of political interest?		The higher the level of political interest, the more comprehensive the HIA should be.		
External interest	What is the level of public interest?		The higher the level of public interest, the more comprehensive the HIA should be.		
External interest	Are there other political & public considerations?		The more complex the considerations, the more comprehensive the HIA should be.		
Timing/ External interest	Is there a 'window of opportunity' for the work?		Consider if there is a window of opportunity (ie. Timeliness, currency, political support). If the window is close, select the less comprehensive tool.		
Capacity (in house)	What is the in-house level of expertise in HIA?		The higher the level of expertise, the more comprehensive the HIA should be.		
Capacity (in house)	What level of staff resources and support are available?		The higher the resource and support level, the more comprehensive the HIA should be.		
Capacity (external)	What level of expert support is available?		The higher the level of expert support, the more comprehensive the HIA should be.		
Resources	What funds are available?		The higher the level of funding, the more comprehensive the HIA should be.		
Resources	What data associated with the proposal is available and accessible? What is the health evidence base associated with the proposal?		If more data is available and accessible, the more comprehensive the HIA should be.		

Supplementary Table 3: Core values and guiding principles

**Value/Principle**	
Democracy	With the goal of informing and influencing future directives, it is important to involve community members in directive reformulation. Democracy accentuates the right to participation in a transparent process of directive reformulation affecting lives and livelihoods (2).
Equity	The goal of the public health direction should be to protect the health of the target population equitably. However, this involves accommodating for differences in the impact of the directive; eliminating factors which may be avoidable and unfair in terms of gender, age, ethnic background and socio-economic status resulting in inequities and furthering health inequalities (2).
Sustainable Development	In implementing the directive, short-term and long-term, direct and indirect impacts of the directive need to be considered.
All-inclusive Protection	Public health directions protecting population health should aim to protect all facets of health, including physical, and mental wellbeing adopting a comprehensive health for all focus.
Socially Just	Public health directives should seek to be socially just and equitable in their implementation and outcomes (2).
Accountability	Decision-makers should be accountable to the communities they serve (2).

Supplementary Table 4: Search terms and combinations used to find evidence

**Search terms and combinations used for literature searches**
COVID19, lockdown, psychological distress, men
COVID19, lockdown, psychological distress, women
COVID19, lockdown, psychological distress, gender difference
COVID19, lockdown, income, gender difference
COVID19, lockdown, occupation, gender difference
COVID19, lockdown, psychological distress, essential worker
COVID19, lockdown, psychological distress, healthcare worker
COVID19, lockdown, loneliness, gender difference
COVID19, lockdown, social isolation, gender difference
COVID19, lockdown, relationship, gender difference
COVID19, lockdown, family, gender difference
COVID19, lockdown, children, father
COVID19, lockdown, children, mother
COVID19, mental health

Supplementary Table 5: Source of information and methods used to obtain it

**Source of information**	**Methods used to obtain information**
Electronic searches were completed using 2 search strategies:<br>1. Searching published literature in databases<br>2. Searching published reports, government documents, government websites and other grey literature using Google	
Published literature databases	Electronic literature searches on PubMed, Google Scholar, Cochrane Library
ABS Census Data	Google search terms: ABS, Victoria, Australia, population data, gender indicators<br>https://www.abs.gov.au/statistics/people/people-and-communities/gender-indicators-australia/latest-release#data-download<br>https://www.abs.gov.au/statistics/people/population/national-state-and-territory-population/latest-release<br>https://www.abs.gov.au/census
Department of Health and Human Services (Victoria) website	Google search terms: Victorian health<br>https://www.dhhs.vic.gov.au/coronavirus<br>https://www.health.vic.gov.au/
Victorian Stay-at-Home Directions	Google search terms: Victoria, Stay-at-home directions<br>Stay-at-home directions are archived when finished therefore not found at one website. URLs for each direction can be found in the reference list.
Government websites	Google search terms: Premier of Victoria, Media releases Victoria Premier of Victoria website, Chief Health Officer Victoria<br>https://www.premier.vic.gov.au/<br>Press/media releases<br>https://www.premier.vic.gov.au/media-centre<br>https://www.health.vic.gov.au/media-centre/media-releases<br>https://www.vic.gov.au/media-events-and-publications<br>Chief Health Officer<br>https://www.health.vic.gov.au/public-health/chief-health-officer
Health and population data (Victoria)	Google search terms: Victoria<br>Informed decisions community demographic resources<br>https://profile.id.com.au/australia/about?WebID=110
Royal Commission into Victoria's mental health	Google search terms: Victoria, mental health<br>https://finalreport.rcvmhs.vic.gov.au/
ABS Household Impact of COVID-19 data	Google search terms: ABS, COVID19<br>https://www.abs.gov.au/statistics/people/people-and-communities/household-impacts-covid-19-survey
Newspaper articles	Google search

Supplementary Table 6: Impact Assessment Matrix (reproduced from Appendix 3: Comprehensive Assessment Matrix, Health Impact Assessment: A Practical Guide (1))

ACTIVITY: LOCKDOWN VICTORIA Stay-at-home-directive (No7) (3)						
POPULATION: WOMEN						
	PSYCHOLOGICAL DISTRESS	HEALTH IMPACT: INCREASED PSYCHOLOGICAL DISTRESS				
		Psychosocial Determinants				
		LONELINESS	SOCIAL ISOLATION	RELATIONSHIPS/ FAMILY LIFE	INCOME	OCCUPATION
SOURCE OF INFORMATION	Large population-based survey, Local state survey, population-based surveys research-based, published studies, preprint studies	Large population-based survey, Stratified, clustered sampling observation, local & government survey, qualitative, prospective cohort study,	Local government populations-based survey and published population-based survey	National government survey, local organisation survey, large population-based surveys	Government surveys, large population-based survey	Government surveys, local hospital data, published papers hospital surveys
STRENGTH OF EVIDENCE	LIMITED DATA<br>MODERATE STRENGTH	LIMITED DATA<br>MODERATE STRENGTH	LIMITED DATA<br>LIMITED STRENGTH	LIMITED DATA<br>MODERATE STRENGTH	LIMITED DATA<br>MODERATE STRENGTH	LIMITED DATA<br>MODERATE STRENGTH
NATURE OF IMPACT	NEGATIVE					
DURATION OF IMPACT	Long term – acute severe distress may transfer to long term mental illness. Short term – mild distress, increase in health risk behaviours	Long term – severe trigger for mental illness. Short term – mild situational, increase in health risk behaviours	Long term – trigger for mental illness Short term – mild situational, increase in health risk behaviours	Long term – trigger relationship breakdown, mental illness<br>Short term – mild situational	Long and short term – increased poverty and social disadvantage. Increase in health risk behaviours.	Long term – burnout, mental illness<br>Short term – work stress.
SIZE OF IMPACT	LARGE – potentially 50% of the entire population					
LIKELIHOOD	Highly probable	Probable	Probable	Probable	Highly probably	Highly probable
POPULATION BEARING DIFFERENTIAL IMPACTS	• Young adults<br>• Low income<br>• CALD<br>• Greater Melbourne<br>• People with a disability<br>• Unemployed<br>• Mothers	• Living alone<br>• Living as sole parents<br>• Older adults<br>• Socially Disadvantaged<br>• Low income<br>• Pregnant women<br>• Younger women	• People with disability<br>• Unemployed<br>• Low income<br>• Regional/remote<br>• Older people<br>• Younger people<br>• CALD<br>• Aboriginal & Torres Strait	• Mothers<br>• Carers<br>• CALD<br>• Aboriginal & Torres Strait<br>• Socially disadvantaged	• Low income<br>• CALD<br>• Socially disadvantaged<br>• People with a disability<br>• Young adults<br>• Aboriginal & Torres Strait	• Healthcare and social care workers

SCOPE FOR RECOMMENDATION ADOPTION	High	Medium	Medium	High	High	Medium

Supplementary Table 6:

References

1. Harris P, Harris-Roxas B, Harris E, Kemp L. Health Impact Assessment: a practical guide. Sydney: UNSW Research Centre for Primary Health Care and Equity and NSW Health. 2007.
2. Mahoney M, Simpson S, Harris E, Aldrich R, Stewart-Williams J. Equity-focused health impact assessment framework. 2004.
3. Public Health Commander. Stay at Home Directions (Restricted Areas) (NO 7) Victoria: Victoria State Government; 2020 [Available from: https://www.dhhs.vic.gov.au/sites/default/files/documents/202008/Stay%20at%20Home%20Directions%20%28Restricted%20Areas%29%20%28No%207%29%20-%202%20August%202020.pdf.
